# Comparison of endoscopic transcanal and microscopic approach in Type 1 tympanoplasty

**DOI:** 10.1016/j.bjorl.2019.07.005

**Published:** 2019-09-04

**Authors:** Secaattin Gulsen, Adem Baltacı

**Affiliations:** a Dr. Ersin Arslan Training and Research Hospital, Department of Otorhinolaryngology, Gaziantep, Turkey; b Private HATEM Hospital, Department of Otorhinolaryngology, Gaziantep, Turkey; c Gölbaşı State Hospital, Department of Otorhinolaryngology, Gölbaşı, Turkey

**Keywords:** Tympanoplasty, Endoscopic ear surgery, Cartilage, Perforation, Transcanal

## Abstract

**Introduction:**

Endoscopic tympanoplasty is a minimally invasive surgery that may be performed via a solely transcanal approach. The use of endoscopes in otologic procedures has been increasing worldwide. The endoscopic approach facilitates the transcanal tympanoplasty, even in patients having the narrow external ear canal with an anterior wall protrusion.

**Objectives:**

The present study aimed to compare the surgical and audiological outcomes of endoscopic transcanal and conventional microscopic approach in Type 1 tympanoplasty.

**Methods:**

The graft success rates, hearing outcomes, complications, and duration of surgery in patients who underwent endoscopic and microscopic tympanoplasty between October 2015 and April 2018 were retrospectively analysed.

**Results:**

Graft success rates were 94.8 per cent and 92.9 per cent for the endoscopic and microscopic group, respectively (*p* >  0.05). Postoperative air-bone gap values were improved significantly in both groups (*p* <  0.001). The average duration of surgery was significantly shorter in the endoscopic group (mean 34.9 min) relative to the microscopic group (mean 52.7 min) (*p* <  0.05). The average hospitalization period was 5.2 h (range 3–6 h) in Group I whereas it was 26.1 h (range 18–36 h) in Group II (*p* <  0.05).

**Conclusion:**

The endoscopic transcanal tympanoplasty approach is a reasonable alternative to conventional microscopic tympanoplasty in the treatment of chronic otitis media, with comparable graft success rates and hearing outcomes.

## Introduction

The main objectives of tympanoplasty include the closure of the tympanic membrane perforation and restoration of hearing loss. To date, alternative surgical approaches, different grafting techniques, and various graft materials (fat, vein, cartilage, fascia, skin) have been used for tympanoplasty.^1^, ^2^, ^3^, ^4^ The use of endoscopes in middle ear surgery began in the 1990s and became widespread across the globe.^3^, ^4^ Presently, endoscopes, as a primary or auxiliary device, are used in almost all kinds of middle ear surgeries such as chronic otitis surgery, stapes surgery, and cochlear implantation.^5^, ^6^ Endoscopes, particularly angled types, provide direct access to the concealed areas which can not be visualized fully without bone curettage via conventional microscopic approaches, such as the anterior epitympanum, retrotympanum, and hypotympanum.^7^ In addition, the endoscopic approach provides significant advantages such as panoramic vision, high image quality, and ease in achieving the desired zoom and exposure by simply pushing the endoscope back and forth.^8^, ^9^ Despite these advantages, one-handed surgery, lack of stereoscopic vision, longer initial operative times, and prolonged learning curve are the limiting aspects of the endoscopic approach.^9^ Since endoscopic transcanal tympanoplasty is a minimally invasive technique, it results an almost painless postoperative period and results in a short period of hospitalization. In this study, the authors intended to compare the surgical and functional outcomes between Endoscopic Transcanal Tympanoplasty (ETT) and Microscopic Tympanoplasty (MT) in patients with tympanic membrane perforation.

## Methods

In this retrospective study, the data of 126 patients who underwent ETT and MT between October 2015 and April 2018 were analysed. This study was carried out in Dr. Ersin Arslan Research and Training Hospital (Tertiary referral centre) and Private Hatem Hospital. All patients were operated by the same surgeon (first author). An informed consent form was obtained from all patients, and local ethics committee approval (reference number: 2018-264) was obtained prior to study conduction. Patients with a history of ear surgery, patients with cholesteatoma who underwent mastoidectomy, and patients who could not be followed adequately were not included in the study. After a minimum of 6 months of follow-up, the grafting was considered to be successful in patients having no graft perforation and medialization. Pure Tone Audiometry (PTA) was performed at frequencies of 500, 1000, 2000 and 4000 Hz to determine the Air Conduction (AC) thresholds, Bone Conduction (BC) thresholds and Air-Bone Gap (ABG) values preoperatively. PTA at the same frequencies was performed again 6 months after surgery. Auditory gain, the difference between preoperative and postoperative ABG values, was calculated and analysed. Perforations of the tympanic membrane were grouped as central, marginal, anterior and posterior with respect to their anatomic locality. Also, perforations of tympanic membrane were classified as small (perforation of the tympanic membrane less than 25%), medium (between 25% and 75%) and large (more than 75%) according to their sizes. Duration of surgery, perforation characteristics (localization and size), graft success rate, complications and hospitalization period were recorded and retrospectively analysed. Our routine follow-up policy included an assessment in the first and second week and at 1, 3 and 6 months after surgery.

### Surgical technique

**Figure 1:**
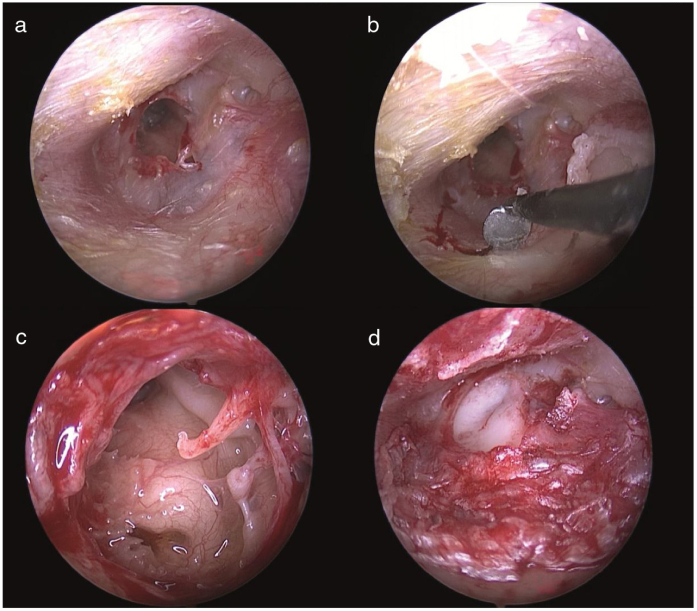
Photographs showing (a) the regeneration of the perforation edges; (b) the transcanal simultaneous incision and cauterization via insulated Rosen blades; (c) an inspection of the middle ear structures; (d) an intra-operative view of the perforation after chondroperichondiral grafting.

**Figure 2:**
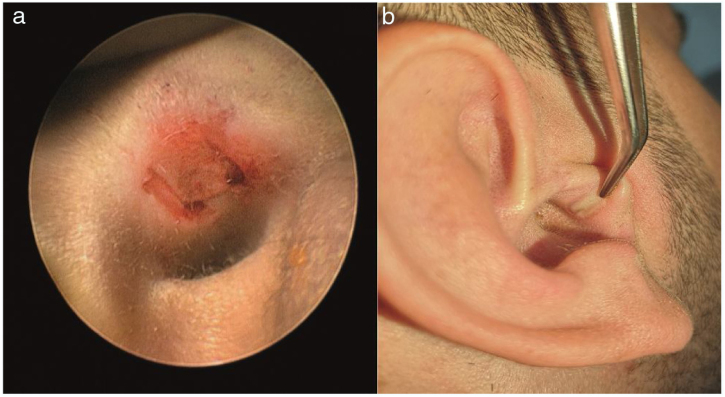
Photographs showing (a) a modified U-shaped graft incision to preserve tragal cartilage rim; (b) an appearance of secondary healed graft incision sixth month after surgery.

In patients undergoing ETT and MT, a chondroperichondrial graft taken from tragus was preferred. A modified U-shaped incision to preserve tragus rim was performed while harvesting a chondroperichondrial graft from tragus. Perforation edges were freshened before transcanal incisions were done. In order to minimize bleeding while making transcanal incisions in the ETT procedure, we used insulated Rosen blades; we touched the tip of the Rosen blade to monopolar cautery and made simultaneous cauterization and tympanomeatal flap incisions. Therefore, we did not experience any significant bleeding during the tympanomeatal flap incision and elevation. The fibrous annulus was separated meticulously from the tympanic sulcus with preservation of the chorda tympani nerve and the middle ear space was reached. Mobility and integrity of ossicular chain were checked by gentle palpation of the ossicles. The notch corresponding to malleus manubrium was created on the chondroperichondrial graft. The chondroperichondrial graft was positioned with the underlay technique (Fig. 1). Lastly, the modified U-shaped incision was tightly supported with Gelfoam® (Pfizer Inc, New York, USA) and left for secondary healing without suturing (Fig. 2). In the MT Group, the retroauricular approach was preferred in all patients. The chondroperichondrial graft with a notch corresponding to malleus manubrium was placed with the underlay technique. Retroauricular incisions were closed by absorbable suturing. A mastoid dressing was applied to the all patients in the microscopic group. In both ETT and MT Group, the tympanomeatal flap was placed in the original position and tightly supported with Gelfoam. Ear drops (Siprogut®, Bilim, Istanbul, Turkey) containing ciprofloxacin were applied to Gelfoam® placed in the external auditory canal.

In the ETT procedure, 0 and 30°-angle rigid endoscopes (Karl Storz Endoscopes, Tuttlingen, Germany), 2.7 mm in diameter and 11 cm in length, were utilized. The endoscopes were connected to an HD camera (Karl Storz, Germany) and the image was transferred to an HD monitor standing in front of the surgeon. An LED light source (Karl Storz, Germany) was used for illumination. Surgical microscope (Möller-Wedel, Haag-Streit Surgical, Rosengarten, Germany) was used in the MT procedures.

### Statistical analysis

The Statistical Package for Social Science (SPSS) 22.0 software was used for statistical analysis of the data. Descriptive and statistical analysis was performed. Normality of data was checked using the Kolmogorov-Smirnov test. Categorical variables between groups were compared using the independent *t*-Test for the graft success rates, duration of surgery, perforations characteristics and auditory gain. The paired *t*-test was used to compare pure tone audiogram results. Results are presented as mean (±Standard Deviation), median (range) and per cent. A *p*-value less than 0.05 was considered to be statistically significant.

## Results

The surgical and functional results of 149 ears of the 126 patients who underwent tympanoplasty with an endoscopic (n = 78) and microscopic (n = 71) approach were analysed. The patients who were operated with the endoscopic and microscopic approaches were partitioned into two groups as Group I (n = 67 patients) and Group II (n = 59 patients), respectively. The average age was 45.4 years (range 15–61 years) and there were 41 (61.1%) males and 26 (38.9%) females in Group I whereas Group II comprises of 28 (47.5%) males and 31 (52.5%) females, and the mean age was 54.8 years (range 18–72 years). ETT was performed on 52 (66.6%) right and 26 (33.4%) left ears in the Group I. In MT Group, 37 (52.1%) of the ears operated were right and 34 (47.9%) were left ears. Demographic characteristics and surgical outcomes of the patients undergoing ETT and MT are presented in Table 1. The majority of perforations in both groups were located in the anterior quadrant of the tympanic membrane. There was no significant difference between groups regarding characteristics of perforations of the tympanic membrane (Table 2). The postoperative mean follow-up period was 8.2 months (range 6–18 months) and 9.3 months (range 8–20 months) in Group I and II, respectively.

**Table 1:** Demographic characteristics and surgical outcomes of the patients.

	Endoscopic group (n = 67)	Microscopic group (n = 59)	*p*-value*
Age (mean-range)	45.4 (15‒61)	54.8 (18‒72)	
Gender			
Male	41 (61.1%)	28 (47.5%)	
Female	26 (38.9%)	31(52.5%)	
Direction			
Right	52 (66.6%)	37 (52.1%)	
Left	26 (33.4%)	34 (47.9%)	
Graft uptake rate (%)	94.8%	92.9%	>0.05
Mean operative duration (mean ± SD)	34.9 ± 11.2	52.7 ± 6.9	<0.05
Mean hospitalization period (hours)	5.2	26.1	<0.05
Mean follow-up duration (months)	8.2	9.3	
Complications			
Hematoma	4 (5.6%)	0	
Wound infection	5 (7.4%)	0	
Wound dehiscence	0	5 (6.4%)	
Dysgeusia	7 (9.8%)	2 (2.5%)	
Otitis externa	8 (11.2%)	1 (1.2%)	
Numbness at auricula	6 (8.4%)	0	
Mild asymmetry at auricula	7 (9.8%)	0	

SD, standard deviation.

* A *p*-value less than 0.05 was considered to be statistically significant.

**Table 2:** Comparison of the perforation characteristics between groups.

	Group I (n, %)	Group II (n, %)	*p*-value*
Perforation localization			
Central	14 (17.9)	15 (21.1)	>0.05
Marginal	12 (15.4)	10 (14.1)	>0.05
Anterior	32 (41.1)	30 (42.3)	>0.05
Posterior	20 (25.6)	16 (22.5)	>0.05
Perforation size			
<25%	42 (53.8)	39 (54.9)	>0.05
25%‒75%	23 (29.5)	22 (30.8)	>0.05
>75%	13 (16.7)	10 (14.1)	>0.05

* A *p*-value <0.05 was considered to be statistically significant.

After a minimum of 6 months follow-up, the graft success rates for Group I (74 out of 78 ears) and Group II (66 out of 71 ears) were 94.8% and 92.9%, respectively; there was no significant difference between the groups in terms of graft success rates (*p* > 0.05). The mean operative time (± Standard Deviation) was 34.9 ± 11.2 min (range 30–51 minutes) and 52.7 ± 6.9 min (range 49–72 min) for patients in Group I and II, respectively. The duration of surgery was significantly shorter in Group I relative to Group II (*p* < 0.05).

When the preoperative and postoperative PTA measurements including AC thresholds and ABG values which are presented in Table 3 are compared, a statistically significant audiologic improvement was observed in both groups (*p* < 0.001). However, no significant difference was found between the Group I and II with respect to average auditory gain (Table 3). Both the endoscopic and microscopic approach are effective in ensuring comparable audiologic outcomes in Type 1 tympanoplasty.

**Table 3:** Comparison of audiological outcomes.

PTA results	Endoscopic group			Microscopic group			p-value*
	Preoperative (mean ± SD)	Postoperative (mean ± SD)	*p*-value	Preoperative mean ± SD)	Postoperative (mean − SD)	*p*-value	
AC (dB)	37.6 ± 4.1	14.6 ± 3.7	<0.001	36.3 ± 7.1	13.6 ± 4.5	<0.001	0.379
BC (dB)	8.2 ± 2.4	7.9 ± 2.1	>0.05	9.5 ± 4.2	9.1 ± 3.9	>0.05	0.189
ABG (dB)	28.9 ± 6.7	8.2 ± 4.7	<0.001	29.7 ± 5.3	7.9 ± 5.7	<0.001	0.256
Average auditory gain (dB)	19.4 ± 5.7			18.7 ± 6.8			>0.05

PTA, Pure Tone Audiometry; AC, Air Conduction; BC, Bone Conduction; ABG, Air Bone Gap; SD, Standard Deviation.

* A *p*-value <0.05 was considered to be statistically significant. *P*-values in bold belong a comparison between the endoscopic and microscopic group.

The average hospitalization period was 5.2 h (range 3–6 h) in Group I, whereas it was 26.1 h (range 18–36 h) in Group II. Since the patients in Group I had less pain and minimally invasive surgery, the average hospitalization period was significantly shorter as compared to patients in Group II (*p* < 0.05).

Minor complications were observed in the microscopic group as follows: 4 (5.6%) patients had a hematoma in the graft donor site area, wound infection in 5 (7.4%) patients, transient dysgeusia in 7 (9.8%) patients, otitis externa in 8 (11.2%) patients, auricularnumbness in 6 (8.4%) patients, and mild asymmetry of the ear in 7 (9.8%) patients has occurred. In the endoscopic group, external otitis in 1 (1.2%) patient, wound dehiscence in 5 (6.4%) patients and temporary dysgeusia in 2 (2.5%) patients was observed. Any major complications such as facial paralysis or sensorineural hearing loss occurred in none of the patients in the endoscopic or microscopic group.

## Discussion

The main objectives of the treatment of chronic otitis media are to repair the tympanic membrane, to eliminate the chronic infection, and, if necessary, to provide integrity and mobility of the ossicular chain via ossiculoplasty or artificial prostheses.^1^, ^2^, ^3^, ^4^, ^5^ Different surgical approaches, artificial prostheses, various graft materials, and grafting techniques have been used for this purpose.^5^, ^6^ Although currently many otorhinolaryngologists prefer the conventional microscopic approach in otologic operations, the use of the endoscope as a primary or auxiliary device in ear surgery has been rapidly increasing in recent years.^7^, ^8^, ^9^ Despite the significant advantages of the microscopic approach, such as providing the ability to work with both hands, offering stereoscopic vision,and requiring a shorter training period, the insufficiency to provide an adequate view of concealed areas in the middle ear without bone curettage or canaloplasty is the most important limitation of the microscopic approach, particularly in patients with narrow and curved external auditory canals.^10^, ^11^, ^12^ The hidden areas in the middle ear space that can not be visualized directly with the surgical microscopes might be accessed easily without bone curettage or canaloplasty employing angled rigid endoscopes. The ETT with lower complication rates has considerable advantages such as being minimally invasive and providing shorter operative times, offering a panoramic view, and ensuring easy access to hidden areas. On the other hand, lack of stereoscopic vision, initially prolonged operative time and single-handed surgery are the limiting aspects of the endoscopic approach in ear surgery.^12^, ^13^

According to the literature review and meta-analysis study conducted by Tseng et al., graft success rates were 85.1% and 86.4% without a significant difference for endoscopic and microscopic tympanoplasty, respectively.^9^ In the articles comparing the endoscopic and microscopic tympanoplasty, the graft success rates in the endoscopic and microscopic approaches were ranging between 83.3%–100% and 82.4%–100%, respectively.^11^, ^12^, ^13^, ^14^, ^15^, ^16^ The present study is consistent with the literature, in that there was no significant difference found between the endoscopic and microscopic group with respect to graft success rates. Obviously, the endoscopic transcanal approach provides comparable graft success rates relative to the microscopic technique in Type 1 tympanoplasty.

The pre- and postoperative ABG values in studies comparing the endoscopic and microscopic Type 1 tympanoplasty were ranging between 28.5–46.4 dB and 18.1‒8.1 dB, respectively. The current studies reported that postoperative ABG values in either endoscopic or microscopic tympanoplasty significantly improved. However, there was no significant difference between the endoscopic and microscopic approach regarding postoperative auditory gain.^12^, ^13^, ^14^, ^15^, ^16^ In our study, postoperative ABG values in both groups improved significantly as compared to preoperative ABG values. Moreover, we did not detect a significant difference between Group I and II regarding postoperative auditory gain (Table 3). In terms of hearing outcomes, ETT is a safe and reasonable alternative to the conventional microscopic retroauricular approach in Type 1 tympanoplasty.

The studies in the literature reported that operative time was significantly shorter in ETT group relative to the MT group and the average duration of surgery was ranging between 36–74.4 min and 69–107 min for endoscopic and microscopic tympanoplasty, respectively.^11^, ^12^, ^13^, ^14^, ^15^, ^16^ In microscopic tympanoplasty procedures frequently retroauricular or endaural approaches are preferred. Therefore, large surgical incisions as compared to endoscopic transcanal incisions and the closure of those are factors that prolong the duration of surgery. Furthermore, microscopic tympanoplasty procedures occasionally require extra time-consuming procedures, such as bone curettage and canaloplasty, in order to surgical correct anterior marginal perforations and in order to access the hidden areas in the middle ear space. Consequently, such procedures in microscopic tympanoplasty may lead to longer operative times. On the other hand, sometimes bothersome and time-consuming bleeding which is difficult to control while operating with one hand may occur at the tympanomeatal flap elevation stage in the ETT procedure. To our knowledge, the present study has reported the shortest average operative time in endoscopic transcanal tympanoplasty as compared relevant studies in the literature yet. In the ETT procedure, while performing transcanal incisions and elevating the tympanomeatal flap, we used insulated surgical instruments as shown in Fig. 1. Therefore time-consuming bleeding has been avoided by simultaneous incision and cauterization. The modified U-shaped graft incision was firmly supported with Gelfoam and left for secondary healing without suturing (Fig. 2). In our study, the use of insulated surgical instruments and sutureless closure of the graft incision are the factors that may contribute to achieving the shortest average operative time reported in ETT. Furthermore, time-consuming maneuvers such as position changing, zooming in/out, and focusing are required in order to obtain the desired image in the microscopic approach. In contrary, there is no need for such maneuvers in the endoscopic approach; the desired image and magnification can be achieved by simply pushing forward and rotating the endoscope without any time loss.

In a prospective randomized controlled study conducted by Kaya et al., the authors reported that postoperative pain was significantly lower in the endoscopic tympanoplasty group than in the group undergoing microscopic tympanoplasty.^13^ Similarly, Chen and Hsieh reported that the patients who underwent endoscopic tympanoplasty had a significantly lower level of pain as compared with microscopic tympanoplasty.^17^ Moreover, since the ETT is a minimally invasive procedure, the hospitalization period of the patients in Group I was significantly shorter than Group II. The patients who underwent endoscopic tympanoplasty were discharged 6th hours at the latest postoperatively; whereas all patients in the microscopic group were hospitalized at least 18 h. In addition, minor asymmetry and numbness at the auricle are the rare but bothersome minor complications seen in retroauricular approaches. Incision- related minor complications including hematoma, numbness at the auricle, wound infection, and asymmetry at the auricle were not observed in any of the patients in the endoscopic group.

Finally, in a pilot study, the authors stated that the use of 3D-endoscopes was highly effective in providing stereoscopic vision in the middle ear and lateral skull base surgeries.^18^ Thus, the lack of depth perception, one of the limiting aspects of endoscopic approaches, will be a no longer restrictive issue. Also, it should be kept in mind that 3D endoscopes are expensive devices and surgeons, especially in developing countries, may not have opportunity to employ them. In our opinion, 3D endoscopes are not cost-effective systems because as the experience in ETT increases the surgeon will be accustomed to working with the two-dimensional view of the standard endoscope in a short period of time.

## Conclusions

The endoscopic transcanal approach in Type 1 tympanoplasty with fewer complication rates and shorter operative times is an innovative approach that yields comparable hearing results and graft success rates when contrasted to the conventional microscopic retroauricular approach. Consequently, the endoscopic approach is an effective, reasonable, and reliable alternative to the conventional microscopic retroauricular approach in Type 1 tympanoplasty.

## Conflicts of Interest

The authors declare no conflicts of interest.
